# High-Frequency Cerebellar rTMS Improves the Swallowing Function of Patients with Dysphagia after Brainstem Stroke

**DOI:** 10.1155/2022/6259693

**Published:** 2022-08-11

**Authors:** Ling-hui Dong, Xiaona Pan, Yuyang Wang, Guangtao Bai, Chao Han, Qiang Wang, Pingping Meng

**Affiliations:** Department of Physical Medicine and Rehabilitation, The Affiliated Hospital of Qingdao University, No. 16, Jiangsu Road, Shinan District, Qingdao, Shandong Province, China

## Abstract

**Objective:**

To explore the efficacy of high-frequency repetitive transcranial magnetic stimulation (rTMS) of the swallowing motor area of the cerebellum in patients with dysphagia after brainstem stroke.

**Methods:**

A total of 36 patients with dysphagia after brainstem stroke were recruited and divided into 3 groups. Before stimulation, single-pulse transcranial magnetic stimulation (TMS) was used to determine the swallowing dominant cerebellar hemisphere and the representation of the mylohyoid muscle. The three groups of patients received bilateral cerebellar sham stimulation, dominant cerebellar rTMS + contralateral sham stimulation, or bilateral cerebellar rTMS. The stimulus plan for each side was 10 Hz, 80% resting movement threshold (rMT), 250 pulses, 1 s per stimulus, and 9 s intervals. Sham rTMS was performed with the coil held at 90° to the scalp. The changes in the motor evoked potential (MEP) amplitude and the clinical swallowing function scales of the patients after stimulation were compared among the three groups.

**Results:**

34 patients were finally included for statistical analysis. The scores of penetration aspiration scale (PAS) and functional dysphagia scale (FDS) of the patients after 2 weeks of rTMS in the unilateral stimulation group and bilateral stimulation group were better than that in the sham stimulation group, and there was no significant difference between the two groups. The increase in the MEP amplitude of the cerebral hemisphere in the bilateral stimulation group was higher than that in the other two groups, and the increase in the MEP amplitude in the unilateral stimulation group was higher than that in sham stimulation group. There was no correlation between the improvement in patients' clinical swallowing function (PAS scores and FDS scores) and the increase in MEP amplitude in either the unilateral stimulation group or the bilateral stimulation group.

**Conclusion:**

High-frequency rTMS in the cerebellum can improve swallowing function in PSD patients and increase the excitability of the representation of swallowing in the bilateral cerebral hemispheres. Compared with unilateral cerebellar rTMS, bilateral stimulation increased the excitability of the cerebral swallowing cortex more significantly, but there was no significant difference in clinical swallowing function.

## 1. Introduction

Dysphagia is one of the most common sequelae of stroke. The incidence of poststroke dysphagia (PSD) is more than 50% [1], which usually leads to complications such as malnutrition, pneumonia, and dehydration [2, 3]. Some stroke patients recover from dysphagia within 2 weeks of its onset. However, many patients still have long-term dysphagia and rely on enteral or parenteral nutrition for survival [4, 5]. Thus, searching for an effective therapeutic method becomes an important task to speed up the recovery of swallowing function and reduce these risks.

Currently, the available treatment methods for PSD are mainly based on compensation technology and physical therapy [6, 7]. Physiotherapy aims to strengthen the muscle groups (facial muscles, suprahyoid muscles) to restore tension, strength, range of motion, speed, and coordination [8]. Although some progress has been made in these treatments, the clinical evidence for PSD treatment is limited [9].

Due to the lack of effective treatments, researchers have begun to explore ways to promote the recovery of swallowing function by enhancing neuroplasticity. Previous neuroimaging studies found that the regional cerebral blood flow of the bilateral cerebellum was significantly increased when healthy subjects swallowed saliva spontaneously, suggesting that the cerebellum may be involved in spontaneous swallowing [10, 11]. In addition, a task-state functional magnetic resonance study found that the cerebellum showed functional connections with the primary motor cortex, inferior frontal gyrus, basal ganglia, and thalamus during swallowing. Its role may be related to the coordination of oral and pharyngeal muscle tissues [12].

Based on the above findings in the field of neuroimaging, Jayasekeran et al. tried to use external force to interfere with the cerebellum to explore its impact on swallowing. Studies have found that stimulating a healthy human cerebellum with a single pulse of TMS can generate pharyngeal contractor motor evoked potentials (MEPs) similar to those found when stimulating the cerebral cortex. It has also been found that the use of repetitive transcranial magnetic stimulation (rTMS) at the target can induce stronger MEPs, which means that cerebellar rTMS may promote swallowing movement [13]. Since the effect of rTMS depends on various stimulation parameters (mode, frequency, intensity, pulse number, etc.) [14], Vasant et al. used different pulse numbers and different stimulation frequencies (5, 10, and 20 Hz) of unilateral cerebellar hemisphere rTMS and found that only 10 Hz stimulation could obviously improve the MEP amplitude of the swallowing cortex in both cerebral hemispheres. The effect was the highest at 250 pulses and could last for at least 30 minutes. Regardless of which side of the cerebellar hemisphere was stimulated, there was no difference in the improvement of excitability of the bilateral cerebral cortex hemispheres [15].

In addition, the study used inhibitory rTMS in the swallowing cortex of healthy volunteers to simulate dysphagia after unilateral cortical stroke. It was found that unilateral and bilateral cerebellar rTMS (10 Hz, 250 pulses) can inhibit the negative behavioural effects caused by cortex suppression and improve cortical excitability. The excitability of the cerebral cortex after bilateral cerebellar rTMS is improved more significantly [16]. Vasant et al. used a case to report the positive effect of cerebellar rTMS on the swallowing function of a patient whose stroke centre was following a right posterior inferior cerebellar artery territory infarction [17]. The reason for this result is unclear, and it may be related to the increased signal afferent to the brainstem by cerebellar rTMS.

However, the credibility of individual case analyses is limited. Thus, we investigated the effect and safety of rTMS on dysphagia patients with brainstem stroke by comparing unilateral, bilateral, or sham cerebellar rTMS stimulation.

## 2. Methods

### 2.1. Participants

**Figure 1:**
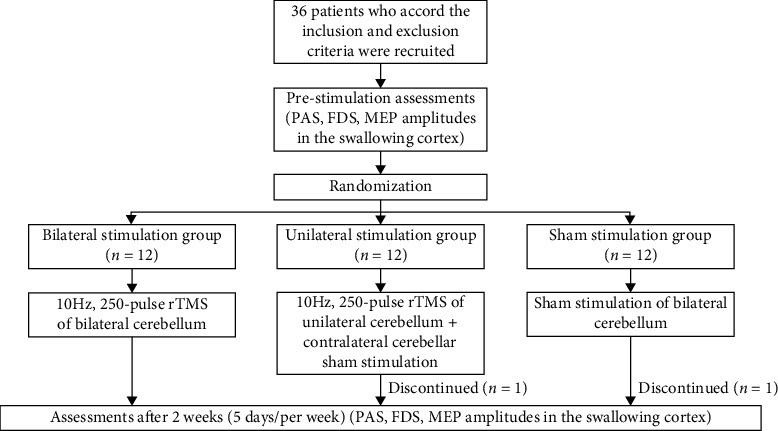
The study design and flow chart.

This study included 36 patients who were hospitalized in the West Coast Ward of the Rehabilitation Medicine Department of the Affiliated Hospital of Qingdao University from May 2020 to May 2021. The inclusion criteria were as follows: patients diagnosed with a brainstem stroke; patients with a duration of the disease less than 6 months; patients with PSD lasting for more than 2 weeks; swallowing disorders confirmed by the videofluoroscopic swallowing study (VFSS). The exclusion criteria were as follows: patients suffering from other diseases that may cause swallowing disorders; patients with combined stroke sites other than the brainstem; patients with an unstable condition; patients with severe cognitive impairment; and patients with contraindications to transcranial magnetic stimulation. This study was approved by the Ethics Committee of the Affiliated Hospital of Qingdao University (QYFY WZLL 26615). All subjects were aware of the study protocol and signed an informed consent form. The enrolled patients were divided into 3 groups using stratified blocked randomization: a sham stimulation group (*n* = 12), a unilateral stimulation group (*n* = 12), and a bilateral stimulation group (*n* = 12). Neither the patients nor the physicians responsible for the evaluation were aware of the distribution of the treatment options in each group. The study design and flow chart are illustrated in Figure 1.

### 2.2. rTMS Protocols

**Figure 2:**
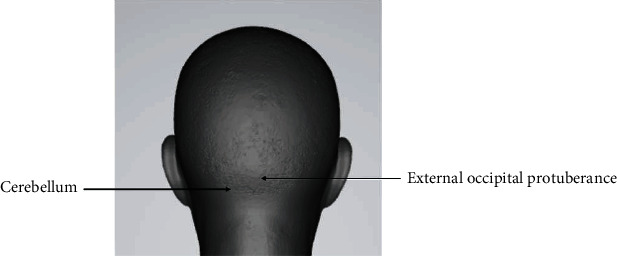
Schematic diagram of the cerebellum.

rTMS was delivered by a magnetic stimulator (Yiruide CCY-IA, Wuhan, China) with a 70 mm circular coil, with a maximum stimulator output of 3.0 Tesla. Before rTMS, the MEP amplitude of the mylohyoid muscle of the bilateral cerebral cortex was recorded. The patients sat in a relaxed position and used alcohol to clean the neck skin, which could remove oil and increase the electrical conductivity between the skin and the electrode. Using a single-pulse TMS system, the coil was tangent to the skull at 45°, and the electromyogram of the mylohyoid muscle was recorded through the surface electrode. The recording electrode was placed 2 cm on the left and right sides of the midpoint of the connection between the mandible and the middle of the hyoid bone. The reference electrode was attached to the mandibular angle. Moving within the area of 2-4 cm in front of the apex of the patient's skull and 4-6 cm from the side, an 80% output was used to obtain the largest motor evoked potential, which is the representation of the mylohyoid muscle of the cerebral cortex. Single-pulse TMS acts on the representation of the mylohyoid muscle motor cortex and gradually reduces the output intensity to determine the rMT. rMT is defined as the lowest TMS intensity of 5 out of 10 trials that can excite the MEP amplitude greater than 50 *μ*v and expressed as a percentage of the maximum output intensity of the stimulator. The average of 5 effective MEP amplitudes was recorded as an index to quantify the excitability of the brain swallowing cortex. The same method was used to find the representation of bilateral cerebellar mylohyoid muscles movement 1 cm below the patient's extraoccipital carina and 3 cm laterally and to determine the rMT and MEP amplitudes (Figure 2), the dominant side with the lower rMT or the higher motor evoked potential amplitude when the rMT was equal. In the unilateral stimulation group, the dominant cerebellum was selected for rTMS, and then, the contralateral side was sham stimulated. In the bilateral group, the dominant side rTMS was performed first, followed by the contralateral side rTMS. In the sham stimulation group, the dominant side sham stimulus was performed first, followed by the contralateral side sham stimulus. The stimulus plan for each side was 10 Hz, 80% rMT, 250 pulses, 1 s per stimulus, and 9 s intervals. Sham rTMS was performed with the coil held at 90° to the scalp. Treatment was provided for a total of 2 weeks, 5 days a week, once a day. The 3 groups of patients received conventional dysphagia rehabilitation performed by a well-trained physical therapist after each rTMS treatment. Traditional swallowing function training included temperature stimulation, air pulse stimulation, taste stimulation, tongue resistance training, and throat lift training, and the training is about 20 minutes after the daily rTMS treatment.

### 2.3. Swallowing Function Assessments

According to the standard manual guidelines, a speech therapist performed the VFSS to assess the patients' swallowing function [18]. The VFSS is the gold standard for evaluating swallowing physiology and is commonly used in clinical settings [19]. In this study, the same protocol for the VFSS in the fluoroscopy laboratory was used for all subjects. Both lateral and posteroanterior images were obtained following oral administration of 5 ml of a thick liquid (fruit pudding) mixed with diluted barium. All materials were standardized. The FDS is a scale used to quantify the severity of dysphagia [20], and the PAS is used to evaluate airway invasion [21]. The FDS and PAS scores were determined by a speech therapist who was not informed about the study and patient grouping according to the VFSS.

### 2.4. Statistical Analysis

IBM SPSS Statistics 22.0 (IBM SPSS, Armonk, NY, USA) was used for the data analysis. Enumeration data were expressed as rates (%), and the chi-square test was adopted. Measurement data were expressed as the mean ± standard deviation (±sd), and the Kolmogorov–Smirnov test was used to determine whether the metrological data followed a normal distribution. Within-group comparisons before and after treatment were performed using paired *t* tests. The independent samples *t* test was utilized for comparisons between two groups. Comparisons among multiple groups were made using one-way analysis of variance with the LSD-t (homogeneity of variance) or Dunnett's T3 (heterogeneity of variance) post hoc test. The correlation analyses were performed using Pearson correlation. The difference was considered statistically significant when the *P* value was less than 0.05 (*P* < 0.05).

## 3. Results

**Figure 3:**
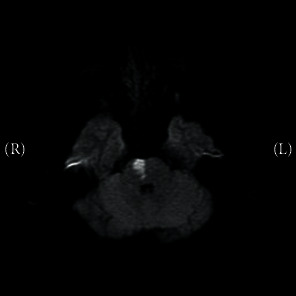
Pontine stroke.

**Figure 4:**
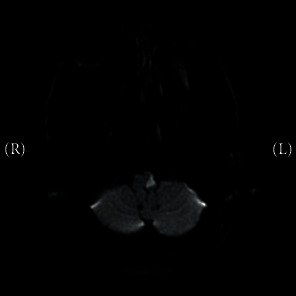
Medullary stroke.

**Figure 5:**
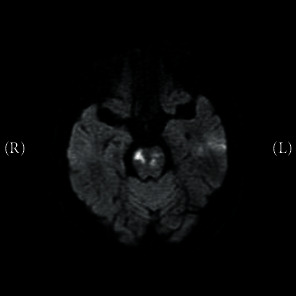
Multiple brainstem stroke.

A total of 36 eligible PSD patients were included in this study. At baseline, the three groups of patients had no significant differences in demographic and clinical characteristics, such as age, sex distribution, disease course, and PAS and FDS scores. During the study period, 1 person in the unilateral stimulation group dropped out due to personal reasons, and 1 person in the sham stimulation group fell off due to personal reasons. Thirty-four patients finally completed the experiment, and details are shown in Table 1. Several representative MRI images showed the lesions in Figures 3–5. Three patients (2 in the bilateral stimulation group and 1 in the unilateral stimulation group) experienced a short-term headache during the treatment, and all recovered within 5 minutes after the stimulation. No patients had seizures during or after the treatment.

### 3.1. Clinical Assessment

**Figure 6:**
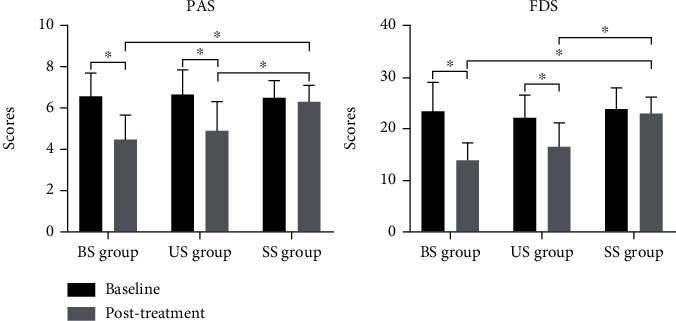
Changes in clinical swallowing function in patients after repeated transcranial magnetic stimulation. BS group: bilateral stimulation group; US group: unilateral stimulation group; SS group: sham stimulation group; PAS: penetration aspiration scale; FDS: functional dysphagia scale. ^∗^*P* < 0.05.

The paired *t* test results showed that the PAS and FDS scores of the patients in the unilateral stimulation group and bilateral stimulation group after 2 weeks of rTMS were better than those before treatment, but there was no significant change in the sham stimulation group. The results of one-way analysis of variance showed that after 2 weeks of treatment, the PAS and FDS scores of the unilateral stimulation group and bilateral stimulation group were better than those of the sham stimulation group, and there was no significant difference between the two groups (Figure 6). This finding indicates that cerebellar rTMS can promote the recovery of swallowing function in patients with brainstem stroke, and there is no significant difference between unilateral stimulation and bilateral stimulation.

### 3.2. Neurophysiological Measurements

**Figure 7:**
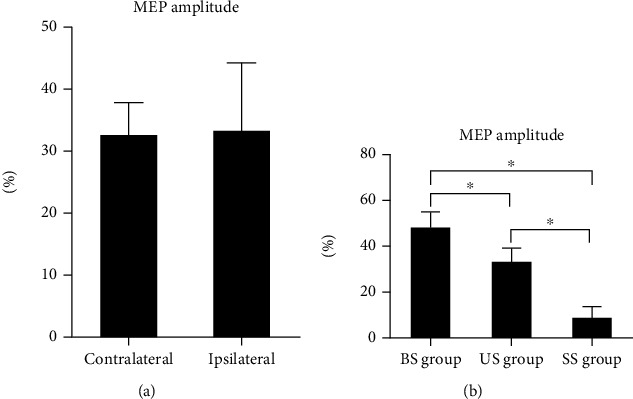
Changes in MEP amplitude in the motor area of the suprahyoid muscle group in the cerebral cortex after treatment. BS group: bilateral stimulation group; US group: unilateral stimulation group; SS group: sham stimulation group; MEP: motor evoked potential. (a) In the US group, the increase in MEP amplitude in the contralateral cerebral cortex (relative to the dominant cerebellum) was not different from that in the ipsilateral cerebral cortex (relative to the dominant cerebellum). (b) The increase in the MEP amplitude of the cerebral hemisphere in the BS group was higher than that in the other two groups, and the increase in MEP amplitude in the US group was higher than that in the SS group. ^∗^*P* < 0.05.

The independent sample *t* test results showed that after 2 weeks of treatment, the increase in MEP amplitude in the contralateral cerebral cortex (relative to the dominant cerebellum) in the unilateral stimulation group was not different from that in the ipsilateral cerebral cortex (relative to the dominant cerebellum) (Figure 7(a)). Since unilateral cerebellar rTMS has no difference in the influence of the MEP amplitude of the cerebral cortex on both sides, the bilateral average elevation amplitude was used for one-way analysis of variance. The results showed that the increase in the MEP amplitude of the cerebral hemisphere in the bilateral stimulation group was higher than that in the other two groups, and the increase in MEP amplitude of the unilateral stimulation group was higher than that of the sham stimulation group (Figure 7(b)). This finding indicates that cerebellar rTMS can improve the excitability of the representative regions of the mylohyoid muscle of a patient's bilateral brain. Unilateral cerebellar stimulation had no significant difference in the effects of the cerebral cortex on both sides, and the effect of bilateral stimulation was higher than that of unilateral stimulation.

### 3.3. Correlation Analyses

**Figure 8:**
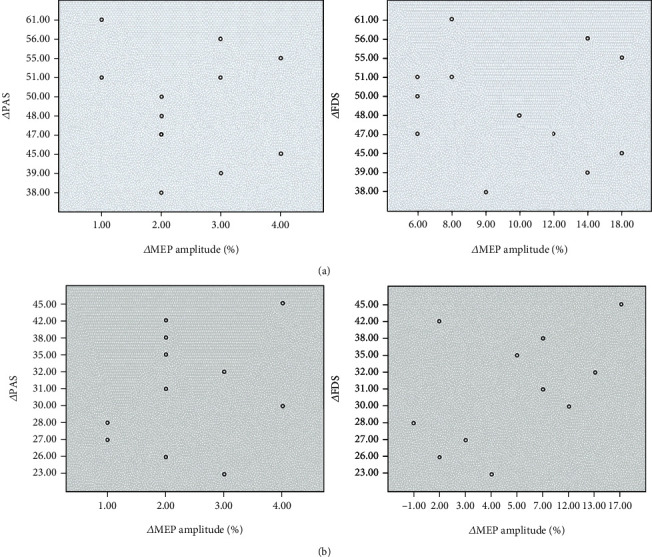
The results of the Pearson correlation analysis showed that there was no correlation between the improvement in patients' clinical swallowing function (ΔPAS: the improvement of PAS after treatment; ΔFDS: the improvement of FDS after treatment) and the increase in MEP amplitude (ΔMEP amplitude: the improvement of MEP amplitude after treatment) in either the unilateral stimulation group or the bilateral stimulation group. PAS: penetration aspiration scale; FDS: functional dysphagia scale; MEP: motor evoked potential. (a) The scatter plot of bilateral stimulation group; (b) the scatter plot of unilateral stimulation group.

The results of the Pearson correlation analysis showed that there was no correlation between the improvement of patients' clinical swallowing function (PAS scores and FDS scores) and the increase in MEP amplitude in either the unilateral stimulation group or the bilateral stimulation group (Figure 8). This finding indicates that the improvement in clinical swallowing function after cerebellar rTMS stimulation may not be directly related to the improvement in MEP amplitude in the representation of the mylohyoid muscle of the brain.

## 4. Discussion

The results of the study showed that, compared with sham stimulation, performing 10 Hz of excitatory rTMS on the mylohyoid muscle motor cortex of the cerebellum for 2 weeks can improve the clinical swallowing function of patients with dysphagia after brainstem stroke. There was no significant difference between unilateral stimulation and bilateral stimulation. Furthermore, regardless of unilateral stimulation or bilateral stimulation, an increase in the excitability of the motor cortex of the mylohyoid muscle was observed, and the increase in the bilateral group was higher than that in the unilateral group. There was no correlation between the improvement in clinical swallowing function and the increase in cerebral cortex excitability. rTMS is a noninvasive stimulation method to promote neurological recovery after stroke. The conclusion means that cerebellar high-frequency rTMS can improve swallowing function in patients with dysphagia after brainstem stroke and increase the excitability of bilateral cerebral swallowing cortex.

Compared to the known cortical swallowing pathways, the cerebellum and its connections to the brainstem and intracortical swallowing centres are poorly understood. Previous electrophysiological studies have shown that there may be simultaneous connections between unilateral cerebellar hemisphere and bilateral cerebral cortical motor areas [15, 16, 22]. Vasant et al. conducted a study of cerebellar rTMS based on different parameters and found that 250 pulses of cerebellar rTMS (10 Hz) resulted in increased excitability of the bilateral swallowing cortex [15]. In addition, Sasegbon et al.'s study found that cerebellar rTMS was able to reverse the MEP effects and behavioural effects of cortical virtual damage inhibition. Consistent with the findings of previous studies, an improvement in the inhibitory effect was observed regardless of whether rTMS was applied to the ipsilateral or contralateral side of the virtual lesion [22]. Sasegbon et al. then further researched this topic and found that compared with unilateral cerebellar rTMS, bilateral cerebellar rTMS led to stronger changes in cerebral cortex excitability [16].

The cerebellum is connected to the brainstem through three pairs of cerebellar peduncles and communicates with various motor nuclei of the brainstem and motor areas of the cortex through these cerebellar peduncles [23]. On the contralateral side, the role of the cerebellum in the upwards transmission of information may be via the dentate nucleus of the cerebellar hemisphere. Efferent axons from the dentate nucleus enter the contralateral motor cortex after passing through the thalamus [24]. On the ipsilateral side, the pathway through which cerebellar rTMS functions may originate from the cerebellar parietal nucleus, which is in contact with components of the central pattern generator (CPG) in the brainstem [23]. CPGs are responsible for controlling swallowing and are closely associated with the bilateral motor cortex [25].

As early as 1998, Hamdy et al. showed that the recovery of swallowing function after unilateral cerebral cortical stroke may be related to the improvement of the function of the uninjured side of the cerebral swallowing cortex [26]. However, the exact mechanisms underlying the recovery of swallowing function after stroke remain unclear. In previous studies of rTMS in the treatment of PSD, the stimulation targets were mostly located in the cerebral cortex. One study focused on the unaffected cerebral cortex and attempted to apply high-frequency rTMS to improve unaffected cortical function to compensate for the affected hemisphere. The results showed that the application of high-frequency rTMS in the unaffected cerebral hemisphere can improve the PAS score of PSD patients, and the treatment effect can be sustained for at least two weeks after the end of treatment [27]. In addition, one study showed that combined stimulation of bilateral cerebral cortex appeared to be more effective than unilateral stimulation. This study demonstrates that 10 Hz bilateral high-frequency rTMS can improve swallowing function in patients with PSD. After 2 weeks of treatment, the patients' swallowing function and risk of aspiration were improved compared with those before treatment, and the effect was better than unilateral stimulation group [28]. Unfortunately, both studies mainly included patients with hemispheric stroke. Few studies have studied brainstem stroke. One study has shown that high-frequency rTMS in the cerebral pharyngeal motor cortex can improve clinical function in patients with dysphagia after brainstem stroke [29]. High-frequency rTMS in the bilateral pharyngeal motor cortex may increase the excitability of corticobulbar projections to the brainstem swallowing nucleus, leading to improved swallowing function. High-frequency rTMS in the cerebellum can also improve cerebral swallowing cortex excitability in healthy volunteers and PSD patients simulated by virtual damage [15, 16, 22]. In addition, the cerebellum is connected to the brainstem through three cerebellar peduncles, which communicate directly with various motor nuclei of the brainstem [23]. Since our correlation analysis found that the recovery of swallowing function in PSD patients was not correlated to the improvement of the excitability of the swallowing motor area of the cerebral cortex, it suggested that the recovery of swallowing function in these patients may be the result of the joint actions of multiple brain regions. We hypothesize that cerebellar high-frequency rTMS can directly and positively affect the brainstem via the cerebellar angle and indirectly excite the brainstem via excitatory effects on the swallowing cortex of the brain, resulting in improved swallowing.

In the present study, we found that both the unilateral cerebellar stimulation group and the bilateral stimulation group had increased excitability of the representation of swallowing in the bilateral cerebral cortex, while the sham stimulation group had no change. The increase in the bilateral group was higher than that in the unilateral group, and this greater excitatory effect may be due to greater stimulus input. Unfortunately, this change was not reflected in the clinical effect. Although the clinical swallowing function scores of the unilateral and bilateral groups were better than those of the sham stimulation group, there was no significant difference between the two groups. This may imply that changes in cortical excitability caused by bilateral stimulation are not sufficient to make a difference in the clinical effect.

Unlike previous studies [28, 30–34], the patients' clinical swallowing function in the sham stimulation group (traditional rehabilitation training) in our study did not improve. In previous studies, few patients with brainstem stroke were included because there was no restriction on the stroke site of the patients. We believe that differences in injury site may lead to different treatment effects. This suggests that dysphagia caused by damage to the swallowing center in the brainstem may be more difficult to recover. This means that rTMS may play a more important role in patients with brainstem injury than in patients with cerebral cortical stroke.

Overall, an earlier study by Vasant et al. identified the optimal parameters for cerebellar rTMS to improve cortical excitability [15]. Sasegbon simulated stroke patients through virtual damage and found that cerebellar rTMS could affect the inhibitory MEP effects and behavioural effects caused by the virtual damage, and the effect of bilateral cerebellar stimulation was higher than that of unilateral stimulation [16, 22]. Our study confirmed that cerebellar rTMS is beneficial for the recovery of swallowing function in patients with PSD; by treating patients with brainstem stroke with rTMS, the excitability of the swallowing cortex in the bilateral cerebral hemispheres can be improved.

## 5. Conclusion

High-frequency rTMS in the cerebellum can improve swallowing function in PSD patients and increase the excitability of the representation of swallowing in the bilateral cerebral hemispheres. Compared with unilateral cerebellar rTMS, bilateral stimulation increased the excitability of the cerebral swallowing cortex more significantly, but there was no significant difference in the improvement of clinical swallowing function. The improvement in the clinical swallowing function of the patients was not correlated with the increased excitability of the swallowing motor area of the cerebral cortex.

## 6. Limitation

Because electrophysiological assessments can only be applied to the swallowing cortex, changes in brainstem function in patients were not assessed in this study. The correlation analysis results showed that there was no significant correlation between the excitability changes of bilateral cerebral cortex and the improvement of swallowing function. This suggests that the effects of cerebellar rTMS on swallowing do not only work by improving the swallowing excitatory cortex of the brain. In future studies, the exact mechanism by which cerebellar rTMS affects swallowing function should be further explored.

## Figures and Tables

**Table 1:** Demographic and clinical characteristics of patients in the three study groups.

	Bilateral stimulation group	Unilateral stimulation group	Sham stimulation group	*P* value
No. of subjects	12	11	11	
Age (years)	49.67 ± 11.28	54.18 ± 10.54	57.55 ± 8.57	0.219
Sex (males : females)	6 : 6	7 : 4	6 : 5	0.815
Type of stroke (ischemia : hemorrhage)	11 : 1	10 : 1	9 : 2	0.821
Site of lesion (pons : medulla oblongata : multiple brainstem stroke)	9 : 2 : 1	8 : 1 : 2	9 : 1 : 1	0.830
Disease course (days)	25.5 ± 9.28	21 ± 5.7	24.91 ± 6.89	0.318
PAS (baseline scores)	6.5 ± 1.17	6.73 ± 1.19	6.55 ± 0.93	0.876
FDS (baseline scores)	24.33 ± 5.85	22.55 ± 4.89	23.36 ± 4.48	0.707

Values are presented as the number or mean ± standard deviation. PAS: penetration aspiration scale; FDS: functional dysphagia scale.

## Data Availability

The data that support the findings of this study are available from the corresponding author upon reasonable request.
